# Acaricidal Efficacy of Plants from Ecuador, *Ambrosia peruviana* (Asteraceae) and *Lepechinia mutica* (Lamiaceae) against Larvae and Engorged Adult Females of the Common Cattle Tick, *Rhipicephalus microplus*

**DOI:** 10.3390/vetsci9010023

**Published:** 2022-01-11

**Authors:** Lucía Guzmán, Jorge Luis Malla, Jorge Ramírez, Gianluca Gilardoni, James Calva, Daniel Hidalgo, Eduardo Valarezo, Catalina Rey-Valeirón

**Affiliations:** 1 Departamento de Ciencias Biológicas y Agropecuarias, Facultad de Ciencias Exactas y Naturales, Universidad Técnica Particular de Loja, San Cayetano Alto s/n, Loja 110107, Ecuador; ltguzmanx@utpl.edu.ec; 2 Titulación en Ingeniería Agropecuaria, Facultad de Ciencias Biológicas y Agropecuarias, Universidad Técnica Particular de Loja, Titulación en Ingeniería Agropecuaria, San Cayetano Alto s/n, Loja 110107, Ecuador; jlmalla1@utpl.edu.ec; 3 Departamento de Química, Facultad de Ciencias Exactas y Naturales, Universidad Técnica Particular de Loja, San Cayetano Alto s/n, Loja 110107, Ecuador; jyramirez@utpl.edu.ec (J.R.); ggilardoni@utpl.edu.ec (G.G.); jwcalva@utpl.edu.ec (J.C.); fdhidalgo@utpl.edu.ec (D.H.); bevalarezo@utpl.edu.ec (E.V.); 4 Laboratorio de Investigación en Parasitología Veterinaria, Intercomunal Coro-La Vela, Universidad Nacional Experimental Francisco de Miranda, Coro 4101, Venezuela

**Keywords:** cattle tick, *Rhipicephalus microplus*, control, efficacy, essential oils, *Ambrosia peruviana*, *Lepechinia mutica*

## Abstract

Control measures against common cattle tick *Rhipicephalus microplus* are of the upmost importance because of considerable, deleterious impact on a farm’s economy. Due to resistance phenomena to synthetic acaricides being a constraint in affected farms, the search for plant derivatives as acaricides has increased dramatically in recent years. In this work, essential oils obtained from two Ecuadorian plants, *Ambrosia peruviana* and *Lepechinia mutica* (EO_Ap_, EO_Lm_), traditionally used as insecticides in indigenous communities, were studied on larvae and engorged females at the parasitic stages of *R. microplus*. Larvae and females were treated with five (0.0625, 0.125, 0.25, 0.50 and 1%) and six concentrations (0.125, 0.25, 0.50, 1, 2 and 4%), respectively, of each EOs_Ap/Lm_. A 98–99% larval mortality was achieved with 0.5% of both EOs_Ap/Lm_. EO_Ap_ inhibited oviposition and egg hatching up to 82% and 80%, respectively, and had an overall efficacy of 93.12%. Efficacy of EO_Lm_ was 72.84%, due to the low influence of EO_Lm_ on reproductive parameters. By steam distillation and GC-MS analysis, γ-Curcumene was identified as the main constituent (52.02%) in the EO_Ap_ and Shyobunol (10.80%) in EO_Lm_. The results suggest that major components of both essential oils should be further studied as promissory acaricides against *R. microplus*.

## 1. Introduction

The cattle tick *Rhipicephalus microplus* Canestrini, 1888 (Acari: Ixodidae) is well known in tropical and semitropical regions because of the harmful effects over the cattle production. These harmful effects result in anemia, decreased productivity, depression of the immune system, damage to hides, overspending due to tick control measures and the morbidity and mortality caused by tick-borne diseases (e.g., babesiosis due to *Babesia bigemina*, *Babesia bovis*) [1]. The life cycle of *R. microplus* includes four developmental stages: egg, larva, nymph and adult. The newly hatched larvae creep upon plants or grass to access the host, seek the flanks, thighs, forelegs and udders for attachment. On a typical host, *R. microplus* spends the rest of the life cycle in the same animal; only the engorged female will leave the host to lay her eggs on the ground. In Ecuador, unlimited use of tick-controlling chemicals has resulted in environmental pollution, milk and meat contamination; resistance development in the tick species has been expressed by veterinarians (no published data) and in published reports [2]. Annual potential losses due to *R. microplus* infections were estimated to be USD 3.24 billion in Brazil [3] and USD 573.6 million in Mexico [4]. Industrial, synthetic acaricides have long been used to effectively control the parasitic stages of the tick; however, these molecules have several disadvantages, including development of tick resistance, destruction of saprophytic species and permanence of residues in the animals and the environment with risks to animal and human health [5]. The intensification of the livestock production requires the use of environmentally friendly compounds to control tick infections.

At the present time, Ecuador is considered to be one of the countries possessing the highest rates of biodiversity in the world; 30% of the Ecuadorian populations are indigenous communities, which maintain their ancestral traditions in the use of natural remedies related to medical applications of plants. It makes this country an ideal place to discover new secondary metabolites, as a result of the chemical investigation of living organisms [6]. Plants accumulate organic substances in significant quantities and concentrations and are a renewable source of these substances; hence, they could be exploited economically and sustainably [7]. The use of plant derivatives in the control of veterinary ectoparasites is an area that holds considerable potential for the future and research into their use in vivo is just beginning [8]. Antimicrobial, insecticide and acaricidal effects of essential oils (EOs) have been exhaustively demonstrated [9,10,11]; numerous studies have confirmed the effect of herbal products on ticks, as larval mortality, reductions in weight, egg-laying, fecundity, and egg viability [12,13,14,15]. Compared to synthetic acaricides, phytoacaricides have several advantages: they are eco-friendly, biodegradable and resistance tends to develop slowly because they are a mixture of several active agents with different mechanisms of action [16]. In addition, plant oils enhanced the toxicity of permethrin when applied in combination with a discrete dose of the synthetic pyrethroid [17]. Hence, investigations on plant derivatives constitute a useful approach because EOs are promising sources of naturally occurring bioactive compounds that show acaricide/insecticidal activities. However, although the cytotoxicity caused by essential oils may be advantageous for killing ticks, the oils are not always harmless. For example, essential oils, including those from *Salvia sclarea* and *Melaleuca quinquenervia,* provoke estrogen secretions, which can induce estrogen-dependent cancers. Some essential oils contain photosensitizing molecules (flavins, cyanin, porphyrins and hydrocarbures), which can cause skin erythema or cancer [18].

*Ambrosia peruviana* Willd belongs to the largest family Asteraceae (Compositae), with more than 1620 genera and 23,600 species of herbaceous plants, shrubs, and trees distributed throughout the world. *Ambrosia peruviana*, also known as Peruvian ragweed or “marco”,”altamisa”, “artemisa” in Ecuador and Peru, is widely used in indigenous traditional medicine to treat rheumathism, menstruation disorders, neurological disturbances and as vermifuge and insecticide [19]. There are 35 natural activities of *Ambrosia peruviana* reported from the Phytochemical Interactions Data Base [20].

*Lepechinia mutica* Benth, also known as “Shalshon”, “turullante”, “casa-casa” by Saraguro communities, is endemic to Loja Province and belongs to the Lamiaceae family that comprises approximately 224 genera; the *Lepechinia* genus includes nine species, among which four are endemic to Ecuador [21,22]. Several species are valued in the horticultural trade and used in folk medicine for the treatment of uterine tumors, stomach ailments, diabetes mellitus control and diarrhea treatment. In particular, the leaves of *L. mutica* are believed to relieve headache and nervous affections and Saraguro reported to be used in veterinary medicine [23]. *Lepechinia mutica* essential oil exhibited moderate in vitro activity against five fungal strains, being especially effective against *Microsporum canis* [23,24]. 

Until now, there have been no reports about the effect of *A. peruviana* or *L. mutica* derivatives on ticks. This work aimed at evaluating the acaricidal effect of the EOs obtained from *A. peruviana* and *L. mutica* on parasitic stages, larvae and engorged adult females, of the common cattle tick *R. microplus*.

## 2. Materials and Methods

### 2.1. Obtention of Engorged Females of R. microplus

One-hundred eighty engorged females of *Rhipicephalus microplus* were collected from a bovine in Punzara location at Loja City, Loja Province, Ecuador (04°02′00″ S, 79°14′00″ W) with unpublished reports on shortening of acaricide application times. Throughout the tick sampling, it was difficult to find animals (a) from the same geographical area under the same tick control management, (b) that were not treated with acaricides for more than 30 days and (c) parasitized by an appropriate number of engorged females. Specimens were kept in plastic flasks with small holes until assays, no more than two days after collection. *Rhipicephalus microplus* females were identified by standard keys [25] and used for the assays. 

### 2.2. Larval Package Test (LPT)

Ten adult engorged females were stuck to the lid of a glass Petri dish with double-sided sticky tape and maintained in an incubator at 29 ± 1 °C and relative humidity (RH) 80% for 20 days to obtain eggs and larvae as by Rey-Valeirón et al. [13]. One hundred larvae of 21 days old were used for LPT as by Stone and Haydock [26] with some modifications reported previously [13]. Five concentrations of *A. peruviana* EO (EO_Ap_) and *L. mutica* EO (EO_Lm_) were used (0.0625; 0.125; 0.25; 0.50 and 1%) diluted in 2% Tween40 (*v*/*v*). The positive control groups (most used commercial acaricides in Ecuador, cypermethrin 15% and amitraz 12.5%), were prepared in distilled water at dilution 1:1000 as recommended by manufacturer. The negative control consisted of distilled water. The percentage of mortality was calculated as Abbott [27]. Mortality (%) in the control group was calculated as number of dead larvae/total number of larvae × 100. The assays were repeated 10 times.

### 2.3. Adult Immersion Test (AIT)

AIT was carried out as by Drummond et al. [28] with some modifications as by Rey-Valeirón et al. [13]. Groups of eight engorged adult females were weighed and immersed for 3 min in 25 mL of each EO_Ap_ and EO_Lm_ dilution (0.125, 0.25, 0.50, 1, 2 and 4% in 2% Tween 40, *v*/*v*). Deionized water was used in negative control group. The positive control groups (cypermethrin 15% and amitraz 12.5%) were prepared in distilled water at dilution 1:1000 as recommended by the manufacturer.

### 2.4. Estimation of Efficacy

To evaluate the acaricidal effect of the EO_Ap_, EO_Lm_ and synthetic acaricides on engorged females and to estimate efficacy, the following formulas were used [29]:

(a) Survival period: number of days the ticks were able to survive after each treatment;
(b) Egg hatching (% EH) = (number of larvae)/(total number of unhatched eggs and larvae) × 100;
(c) Inhibition of oviposition (IOv) = (weight of treated females/weight of control females) − (weight of eggs laid in treated group/weight of eggs laid in control group);
(d) Reproductive efficiency (% RE) = (weight of eggs/weight of females) × egg hatching
(e) Efficacy of essential oil/synthetic acaricides = (RE control group − RE treated group/RE control group) × 100.

### 2.5. Statistical Analysis

Data obtained from the experimental design in LPT and AIT were evaluated by analysis of variance (ANOVA) (α = 0.05). The effects included in the ANOVA were EOs, concentrations and the interactions EO × concentration for each parameter. 

LC_50_ y LC_90_ (concentration necessary for 50% and 90% larval lethality, respectively) of essential oils were obtained by a non-linear regression analysis of percentage of larval mortality versus log concentration. The model established by the program (Prism v9.3.0 for Windows, GraphPad Software, San Diego, CA, USA) was based on equation Y = basal mortality value + (maximal mortality value/(1 + 10^ ((LogLC-X) × HillSlope)). 

### 2.6. Obtention and Characterization of Essential Oils

#### 2.6.1. Plant Material

The collection of *A. peruviana* was performed in Los Operadores (4°00′09.8″ S, 79°15′02.2″ W), canton Loja and *L. mutica* in the canton Quilanga (4°17′43.7″ S, 79°23′59.7″ W) both in the province of Loja, Ecuador. The botanical specimens were identified by Dr. Bolivar Merino, at the herbarium of the Universidad Nacional de Loja, Ecuador. Voucher specimens are preserved in the Herbarium of the Universidad Técnica Particular de Loja, Ecuador, under codes HUTPL-5132 for *A. peruviana* and PPN-LA-005 for *L. mutica*, respectively.

#### 2.6.2. Distillation of the Volatile Fraction

The fresh aerial parts of *A. peruviana* (9 kg) and leaves (5 kg) of *L. mutica* were steam distilled immediately after collection in a separate stainless steel Clevenger-type apparatus for 4 h. After distillation, each organic layer was separated from the aqueous phase, dried over anhydrous sodium sulfate and weighted. 

#### 2.6.3. Physical Properties of EOs

The density and refraction index of essential oils were determined according to the standard AFNOR NF T 75-111 and AFNOR NF T 75-112, respectively, as by Ramirez et al. [23]. The procedures were repeated three times and all measurements were performed at 20 °C.

#### 2.6.4. Qualitative Analysis of the EOs

The qualitative analysis of the EOs was performed by GC-MS by injecting 1 µL of each distilled fraction, 1% (*v*/*v*) diluted in cyclohexane. The injector was kept at 220 °C, operating in split mode with a split ratio of 40:1. The carrier gas (He) was set at a constant flow of 1 mL/min. The analysis was performed in thermal gradient conditions, with the following temperature program: 60 °C for 5 min, increased to 110 °C at a rate of 5 °C/min, then to 148 °C at a rate of 2 °C/min. and to 250 °C at a rate of 20 °C/min, then hold at 250 °C for 2.4 min. The MS was operated in SCAN mode, with a scan rate of 2 scan/s within a mass range of 40–350 *m*/*z* at 70 eV. For each chromatographic peak, the corresponding linear retention index (LRI) was calculated according to Van den Dool and Kratz [30], with reference to a mixture of a homologous series of n-alkanes, from nonane to heptadecane and a homologous series of hydrocarbons C10-C25 (TPH-6RPM of CHEM SERVICE), which were analyzed by GC under the same conditions for essential oil. A non-polar capillary column, DB-5 ms 5%-phenyl-methylpolysiloxane, 30 m × 0.25 mm, thickness 0.25 μm film, was used. Samples were dissolved in dichloromethane.

#### 2.6.5. Quantitative Analysis of the EOs

The quantitative analysis of the EOs was performed by GC-FID (Agilent Technologies chromatograph, model 6890N series), with the same instrumental configuration and method of the qualitative analysis. The constituents were quantified by external calibration, using n-nonane as the internal standard. An isomer was used to quantify isomeric metabolites; if an isomer was not available, a structurally close related terpene was selected [31]. Hence, the following terpenoids (purity > 98%) were used as calibration standards: limonene for aliphatic monoterpene hydrocarbons (R^2^ = 0.9962), p-cymene for aromatic monoterpene hydrocarbons (R^2^ = 0.9986), linalool for monoterpene alcohols (R^2^ = 0.9956), carvone for monoterpene ketones (R^2^ = 0.9958), cedrene for sesquiterpene hydrocarbons (R^2^ = 0.9998) and nerolidol for sesquiterpene alcohols (R^2^ = 0.9997). All calibration curves were built on six points. Quantitative results were reported as main values and standard deviations of three distillations of each plant. The percentage content of each oil component was computed from the corresponding GC-FID peak area without applying any correction factor. The analytical parameters were the same as the GC-MS analysis [23].

## 3. Results

### 3.1. Effect of EOs_Ap/Lm_ on Biological and Reproductive Parameters of R. microplus 

#### 3.1.1. Effects on Larvae

**Figure 1:**
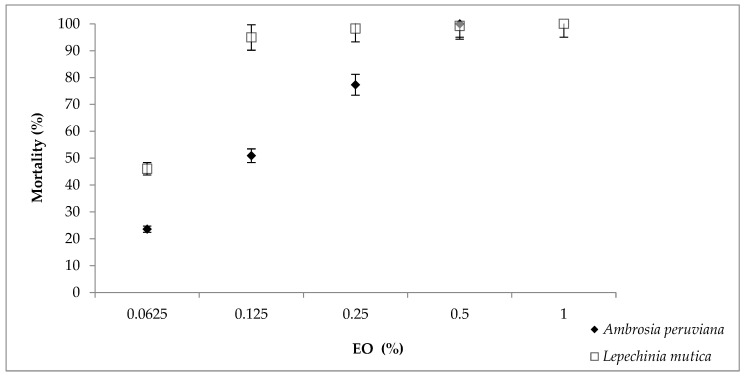
Mortality of *Rhipicephalus microplus* larvae treated with essential oils of *Ambrosia peruviana* or *Lepechinia mutica.* Results are presented as mortality values of 10 replicates as Abbott [27] ±5% error.

The highest value of larval mortality (100%) was achieved with 0.5% EO_Ap_ and 1% EO_Lm_ (Figure 1). At low concentrations (0.0625% and 0.125%), EO_Lm_ caused higher levels of mortality (46% and 95%) compared with EO_Ap_ (24% and 51%); however, a non-lineal trend was observed in both EOs. Statistical differences were not found between 0.25, 0.50 and 1% of EO_Lm,_ (*p* > 0.05) in contrast to EO_Ap_ in which significant differences were obtained between all doses. EOs and control groups were also different (*p* < 0.05). The LC_50_ and LC_90_ values obtained from the results in LPT with EO_Ap_ were 0.118% and 0.388% (R^2^ = 0.623) and with EO_Lm_, 0.063% and 0.118% (R^2^ = 0.592), respectively (Figures S1 and S2). Means and standard errors are shown in Table S1.

Cypermethrin and amitraz (positive controls) in dilution 1:1000 caused 17.59 and 51.41% larval mortality, respectively (*p* > 0.05). The mortality value in control group treated with water was 0.23%. 

#### 3.1.2. Survival Period of Engorged Females

The average survival period of engorged females treated with EOs was dependent on the EO_Ap/Lm_ concentration (Table 1). None of the EO_Ap/Lm_ killed the ticks immediately after the treatment. However, at 4% concentration of EO_Ap_, all the ticks died around four days (4 ± 1.69); with identical concentration of EO_Lm_, the survival period was 9 ± 4.59; with the lower concentration (0.125%) of both EOs, the value was comparable to that of the control group. In general, the ticks survived longer in the group treated with EO_Lm_. Ticks treated with amitraz had a similar survival period (13 ± 8.07) to the 1% EO_Lm_-treated group (13 ± 5.33), but in those treated with cypermethrin (17 ± 3.78), this period was analogous to that of the control group (17 ± 2.53).

#### 3.1.3. Effects on Reproductive Parameters of Engorged Females of *R. microplus*

The effects of EO_Ap/Lm_ on reproductive parameters of *R. microplus* are shown in Table 2. The inhibition of oviposition (IOv) values ranked between 26.71 (0.125%) and 82.27% (4%) in groups treated with EO_Ap_. The optimal value of IOv (82.27%) influenced the lowest value of reproductive efficiency (RE) (3.47%).

The 4% EO_Lm_ inhibited oviposition in 37.62% and RE resulted in 18.97% (*p* < 0.05); concentrations below 4%, although with significant differences (*p* < 0.05), had no relevant effect. Cypermethrin inhibited oviposition in 43.33% and amitraz in 79.58% (*p* < 0.05).

The efficacy of the EO_Ap/Lm_ on the egg hatchability (EH) of *R. microplus* was also evaluated (Table 2). EH (%) with EO_Ap_ ranged between 19.90 (4%) and 88.52 (0.125%), which means that hatching was inhibited up to 80% with 4% of EO_Ap_. 

EH (%) ranged between 60.18 and 91.22 with EO_Lm_. In the control group, the EH value was 94.85%. Results with EO_Ap_ were significantly different to those of the negative control (*p* < 0.05), but not with EO_Lm_, except for the group treated with the concentration at 4%. The EH (%) value with amitraz was 25.60 and with cypermethrin 54.29% (*p* > 0.05). 

As a consequence of the IOv and mortality in EOs groups, RE decreased substantially (Table 2). Lowest RE values (3.47 and 18.97%) were achieved with 4% EO_Ap_ and EO_Lm_, respectively. The RE of control group was 50.44%. No significant differences in RE values were found between concentrations of 0.125, 0.25 and 2% of EO_Lm_ and the control group. While remarkably low RE values were evidenced with both EOs at 4%, the RE was noticeably affected with EO_Ap_ if compared to an identical concentration of the EO_Lm_ (3.47 versus 18.97). Regarding the synthetic acaricides, no statistical differences were found (*p* > 0.05).

The highest efficacy (93.12%) on engorged females was achieved with 4% EO_Ap_; a moderate value was also obtained with 2% EO_Ap_ (70.67%). The efficacy of EO_Lm_ was not as good as those with EO_Ap_. At the higher concentration (4%), EO_Lm_ efficacy was 72.84%; the other concentrations gave negligible values (Table 2). Significant differences were found between both EOs (*p* < 0.05).

### 3.2. Chemical Analysis of Essential Oils from A. peruviana and L. mutica 

#### 3.2.1. Physical Properties of Essential Oils 

The EO_Ap_ was a viscous liquid of subjective color light yellow. The EO_Ap_ yield was 0.06 ± 0.01% (*w*/*w*), referred to fresh plant material. The essential oil relative density was d20 = 0.8872 ± 0.0020 and refraction index was n20 = 1.4963 ± 0.0011. In the leaves of EO_Lm_, d20 = 0.916 ± 0.026, n20 = 1.4867 ± 0.0009, [α] D20 = −5.8 (neat). The EO_Lm_ yield was 0.40 ± 0.12% (*w*/*w*).

#### 3.2.2. Chemical Analysis of the Volatile Fraction 

The qualitative and percent compositions are reported in Appendix A (Table A1). Aromatic sesquiterpene hydrocarbons (57.08%) dominated the characterization of the 41 individual compounds found in the essential oil of *A. peruviana*, with a low amount of aliphatic sesquiterpene hydrocarbons (14.03%), aliphatic monoterpene hydrocarbons (10.83%) and monoterpene ketones (5.57%) to account 92.08% of the total essential oil. The main constituents of the oil were γ-Curcumene (52.02%), Chrysanthenone (5.57%) and ar-curcumene (5.06%). 

In the essential oil distilled from leaves of *L. mutica*, 78 compounds, representing 92.14% the total oil sample. The principal groups of compounds belong to aliphatic sesquiterpene hydrocarbons (35.80%) and aliphatic monoterpene hydrocarbons (24.5%). The most abundant components were Shyobunol (10.80%), δ-3-Carene (8.69%), δ-Cadinene (6.96%), Globulol (5.91%), (E)-Caryophyllene (4.55%), limonene (3.79%) and β-Pinene (3.78%).

## 4. Discussion

The findings in LPT revealed the acaricidal effect of EOs from *A. peruviana* and *L. mutica* on larvae of *R. microplus*. This is the first report of such effect of EOs obtained of both species. The results in the larval package test are of the utmost importance because larvae are considered as strategic targets in the tick control systems. Several authors reported high levels of larval mortality with low concentrations of EOs and it is known that larvae are more susceptible than adults. Mexican oregano (*Lippia graeolens* Kunth) and garlic (*Allium sativum*) EOs at concentration of 1.25% produced high mortality (90–100%) on 10-d-old *R. microplus* tick larvae [16]; cumin seeds (*Cuminum cyminum*) also produced 100% mortality with identical concentration [32]. EOs from *Bursera graveolens* and *Schinus molle* caused 100% mortality of *R. microplus* larvae at 2.5% [14]. However, not all the EOs are effective at low concentrations; for example, *Tetraenia riparia* at 25% or 10% of *Eucalyptus staigeriana* were necessary to achieve a larval mortality of 100% [33,34]. The acaricidal effect may vary because it depends not only on the tick stage but also on the compounds present in the essential oils, which in turn, may show an antagonistic or synergistic effect on potential targets in the arthropod. 

An acaricide that kills females before the third day can prevent egg-laying, disrupting the tick’s life cycle [35] because engorged females begin laying eggs on day 3 or 4 after dropping from the host. In this work, none of the EO_Ap/Lm_ killed the female ticks immediately after the treatment; however, all the ticks died around day 4 (±1.69) in 4% EO_Ap_. Essential oil from *Citrus limonum* exhibited 90% of mortality on engorged females tested after 48 h, reaching 100% at day 16, but these mortality values were obtained with a higher concentration, 10% [36]. 

When the essential oil presents a worthy effect on egg production or hatching, it means a reduction of the RE and a better efficacy when compared to the control groups. In this work, EOAp inhibited the oviposition more than 80%. Ribeiro et al. [37] found that EO from *Hesperozygis ringens* (which has a content of 86.0% oxygenated monoterpene Pulegone), inhibited egg laying by 76.4% at concentration of 5%; *Piper nigrum* and *Pelargonium roseum* EOs at identical concentration inhibited oviposition by 83% and 87.5%, respectively [35]. Those results suggest that sub-lethal doses of essential oils may have an effect on tick fecundity.

Inhibition of EH by EOs has been reported previously. Inside the female tick, the newly formed eggs were not in contact with the EO but there should be a different mechanism of action on the treated engorged female that impairs the viability of the eggs and the subsequent hatching of the larva. Vendramini et al. [38] reported that the andiroba (*Carapa guianensis*) oil acted as an inhibitor of the synthesis and/or incorporation of protein in oocytes of *Rhipicephalus sanguineus*. Because protein is needed to assure viability of the embryo, a reduction in the protein content will lead to the impairment of the embryonic development. Ovicidal effects would reduce the infection of the pastures with newly hatched larvae and thereby decrease cattle infections with the tick.

The results obtained with synthetic, industrial acaricides require further revision due to the low impact on larval mortality and the moderate efficacy on reproductive parameters. Both cypermethrin and amitraz are widely used in cattle farms in Ecuador; resistance values of 67% to amitraz and 50% to alpha-cypermethrin were found in 12 cattle farms [2]. 

In this work, aromatic sesquiterpene hydrocarbons dominated the characterization of the compounds in the EO_Ap_. Both γ-Curcumene and ar-curcumene accounted for more than 57% of the total compounds. Such high percentages of curcumene has only been found in the genus *Senecio* (Asteraceae) [39]. Curcumene as major compound of EOs has been reported as larvicide against two-spotted spider mite, *Tetranychus urticae* [40] and in sand fly tick, *Ornithodoros savignyi* [41]. Shyobunol was the major constituent of the EO_Lm_; the compound has been reported as an important element of the essential oil of *Schinus molle* leaves in Mexico and Tunisia [42], but is not a common major component in plant derivatives. 

Several molecular studies have revealed the action mechanisms of plant-derived EOs that show the efficiency of pesticidal and insect repellent activity and identification of terpenoids as responsible for most of these biological properties [43]. In arthropods, the group of biogenic amine messengers consists of dopamine, tyramine, octopamine, serotonin and histamine; octopamine is an important hormone associated with the nervous system of insects and functions as a neurotransmitter and as a neuromodulator. The inhibition of octopamine will cause the impairment of physiological modulation associated with muscle juncture. Terpenoids are agonists of all types of octopamine and tyramine receptors [43,44]; the study of the effects of EOs on insect-specific octopamin receptor and other potential targets in the tick nervous system should be considered in the quest of candidates for the control of *R. microplus*. 

Together, the results obtained in the present study show promising research in acaricidal effects of components mainly based on EO_Ap_; evaluation of the major components of EO_Lm_ should not be discarded because of a lower LC_50/90_. However, due to the limited number of engorged females used in the present study, it is necessary to confirm the results obtained with both EOs by immersion tests. As the cattle remains infected by ticks at different life stages, the best compound would be that which is effective against all of them. 

The use of botanically-based compounds to control *R. microplus* seems to be a viable alternative, given the number of plants with compounds with activity against the ticks that have already been found, but many factors have to be considered before obtaining a competitive acaricide from *A. peruviana* or *L. mutica*. Although botanicals tend to be less toxic to mammals and other non-target organisms, and their residues are rapidly biodegraded, field validation of experimental formulations and the availability of an adequate delivery system are some of the hurdles for their commercialization [45]. Regardless of all the observations that can be made with the use of essential oils at the field, the excellent level of efficacy obtained with EO_Ap_ at a concentration of 4% cannot be ruled out. This oil deserves further investigation. Both *A. peruviana* and *L. mutica* plants are widely distributed in Loja Province, Ecuador, in areas where there is neither arable soil nor cattle breeding, so these lands are not used for food crops.

Before the advent of synthetic insecticides, nicotine, pyrethrum, and inorganic chemistries dominated the domestic and agricultural pest control arsenal. Pyrethrum, the oleoresin extracted from the dried flowers of the pyrethrum daisy, *Tanacetum cinerariifolium*, led to the synthesis of permethrin, one of the most used acaricidal compounds in the control of cattle tick. Research on plant bioactive compounds can lead to the identification of new molecules presenting different modes of action, biological targets and synergy in comparison to current commercial products [46]. Major compounds could be a reliable alternative to avoid the high costs of EOs extraction and to develop new acaricides. The isolated compound may act as a lead compound or prototype for the synthesis or semi synthesis of pesticide derivatives, which may result in more effective and safer products. However, it should be noted that the isolated compound may promote no activity at all because EO is more effective due to the synergic effect of compound mixture [47]. 

Finally, the results obtained in the present work contribute to the growing list of scientific evidence about the therapeutic efficacy and absence of toxicity in Ecuadorian medicinal plants and their products, as reported recently [48].

## Figures and Tables

**Table 1:** Survival period of *Rhipicephalus microplus* females treated with essential oil of *Ambrosia peruviana* or *Lepechinia mutica*.

Concentration of EO (%)	Survival Period (Days) of Engorged Females	
	*Ambrosia peruviana*	*Lepechinia mutica*
	$\bar{x}$ (sd)	$\bar{x}$ (sd)
4	4 (1.69)	9 (4.59)
2	6 (4.38)	12 (4.64)
1	8 (4.71)	13 (5.33)
0.5	7 (7.75)	14 (4.03)
0.25	9 (4.06)	16 (1.85)
0.125	10 (5.25)	16 (2.50)
Control	17 (2.53)	
Amitraz 1:1000	13 (8.07)	
Cypermethrin 1:1000	17 (3.78)	

**Table 2:** Reproductive parameters in treated groups and efficacy of *Ambrosia peruviana* and *Lepechinia mutica* essential oils on engorged females of *Rhipicephalus microplus*.

EO (%)	IOv (%)		EH (%)		RE (%)		Efficacy	
	*Ap* ^ab^	*Lm* ^ab^	*Ap* ^abc^	*Lm*	*Ap* ^c^	*Lm* ^c^	*Ap* ^ab^	*Lm* ^ab^
0.125	26.71 (18.19)	10.34 (24.25)	88.52 (6.64)	91.22 (19.49)	37.26 (7.43)	51.16 (18.38)	26.38	1.04
0.25	52.27 (39.32)	7.59 (7.70)	56.04 (38.30)	97.59 (3.87)	22.76 ^ab^ (20.95)	48.62 ^ab^ (6.93)	66.42	3.76
0.5	63.19 (16.53)	32.93 (36.75)	39.35 (34.01)	71.20 (44.30)	11.30 ^ab^ (11.69)	31.74 ^ab^ (21.29)	80.90	40.11
1	52.75 (36.44)	26.28 (29.28)	54.79 (46.85)	68.84 (44.23)	23.17 (21.83)	30.83 (19.78)	67.39	38.52
2	65.16 (38.88)	17.57 (21.85)	34.96 (48.03)	86.18 (34.86)	14.79 ^ab^ (21.05)	39.24 ^ab^ (18.07)	70.67	23.73
4	82.27 (18.22)	37.62 (23.94)	19.90 (30.92)	60.18 ^c^ (42.45)	3.47 ^ab^ (5.17)	18.97 ^ab^ (23.74)	93.12	72.84
Control EO	n/a		94.85 (7.90)		50.44 (9.92)		n/a	
Cypermethrin	43.33 (21.00)		54.99 (43.36)		22.66 (23.08)		38.20	
Amitraz	79.58 (23.36)		25.60 (35.62)		8.99 (15.95)		82.15	

EO (%): concentration of essential oil; IOv: inhibition of oviposition; EH: egg hatching; RE: reproductive efficiency; n/a: not applicable; ^a^ Significant differences between concentrations of EO (*p* < 0.05). ^b^ Significant differences between concentrations of EO_Ap_ and EO_Lm_ (*p* < 0.05). ^c^ Significant differences between concentrations of EO and control group.

## Data Availability

The data presented in this study are available on request from the corresponding author.

## Supplementary Materials

The following supporting information can be downloaded at: https://www.mdpi.com/article/10.3390/vetsci9010023/s1. Figure S1. Graph and data for calculation of LC50 and LC90 in larval package test with essential oil of *Ambrosia peruviana*.Figure S2. Graph and data for calculation of LC50 and LC90 in larval package test with essential oil of *Lepechinia mutica*. Table S1. Mean and standard error (SE) of larval mortality values obtained by larval package test with essential oils of *Ambrosia peruviana* and *Lepechinia mutica.*

## Appendix A

**Table A1:** Chemical composition of the essential oil from *Ambrosia peruviana* and *Lepechinia mutica*.

LRI ^a^	LRI ^b^	Compounds	*Ambrosia peruviana*		*Lepechinia mutica*		Type
			%	σ	%	σ	
906	906	Santolina triene	1.22	0.22	-	-	*AMH*
921	926	Tricyclene	-	-	Trace	-	*AMH*
924	924	Thujene <α->	0.35	0.03	Trace	-	*AMH*
931	932	Pinene <α->	0.50	0.04	1.23	0.89	*AMH*
946	949	Camphene	-	-	0.75	0.80	*AMH*
948	945	Fenchene <α->	1.60	0.35	-	-	*AMH*
971	969	Sabinene	-	-	0.24	0.15	*AMH*
974	983	Oct-3-en-1-ol	-	-	Trace	-	*OTH*
976	974	Pinene <β->	-	-	3.78	1.76	*AMH*
988	988	Myrcene	2.91	0.85	0.52	0.28	*AMH*
1003	1003	Mentha-1(7),8-diene <*p*->	-	-	0.16	0.13	*AMH*
1006	1002	Phellandrene <α->	1.40	0.31	3.8	1.70	*AMH*
1008	1008	Carene <δ-3->			8.69	4.24	*AMH*
1016	1014	Terpinene <α->	-	-	0.11	0.07	*AMH*
1019	1020	Cymene <*p*->	0.24	0.16	0.10	0.06	*ARM*
1025	1023	Sylvestrene	-	-	0.29	0.18	*AMH*
1029	1024	Limonene	0.75	0.27	3.79	2.18	*AMH*
1026	1026	Cineole <1,8->	0.31	0.18	-	-	*OTH*
1029	1030	Phellandrene <β->	0.15	0.01	-	-	*AMH*
1045	1044	Ocimene <(*E*)-β->	0.66	0.02		-	*AMH*
1052	1054	Terpinene <γ->	0.38	0.04	0.23	0.12	*AMH*
1065	1071	*cis*-Sabinene hydrate	-	-	Trace	-	*MOH*
1080	1085	Mentha-2,4(8)-diene <*p*->	-	-	0.35	0.18	*AMH*
1084	1086	Terpinolene	0.91	0.59	0.60	0.33	*AMH*
1084	1086	*trans*-Linalool oxide	*-*	-	Trace	-	*MOH*
1095	1102	Linalool	-	-	0.20	0.09	*MOH*
1110	1109	Oct-1-en-3-yl acetate			1.37	0.60	*OTH*
1124	1124	Chrysanthenone	5.57	1.88	-	-	*MKE*
1141	1145	Camphor	-	-	Trace	-	*MKE*
1165	1172	Borneol	-	-	0.25	0.05	*MOH*
1174	1180	4-Terpineol	-	-	0.14	0.02	*MOH*
1194	1186	Terpineol <α->	-	-	0.11	0.02	*MOH*
1283	1284	Bornyl acetate	1.59	0.42	2.20	1.04	*OTH*
1335	1328	Elemene <δ->	Trace	-	Trace	-	*ASH*
1345	1345	Cubebene <α->	0.47	0.26	0.57	0.08	*ASH*
1373	1373	Ylangene <α->	-	-	0.15	0.05	*ASH*
1374	1362	Isoledene	0.33	0.11	-	-	*ASH*
1374	1367	Copaene <α->	-	-	1.46	0.23	*ASH*
1381	1387	Bourbonene <β->	-	-	0.47	0.25	*ASH*
1385	1382	Modheph-2-ene	-	-	-	-	*ASH*
1392	1387	Cubebene <β->	0.56	0.15	0.15	0.04	*ASH*
1405	1410	Cedrene <α->	0.11	0.04	-	-	*ASH*
1407	1409	Gurjunene <α->	-	-	1.94	0.37	*ASH*
1407	1418	Longifolene	-	-	0.15	0.07	*ASH*
1417	1411	Funebrene <2-*epi*-β->	-	-	Trace	-	*ASH*
1417	1412	(*E*)-Caryophyllene	2.01	0.11	4.55	2.16	*ASH*
1419	1429	Thujopsene <cis->	0.26	0.01	-	-	*ASH*
1424	1431	Copaene <β->	-	-	0.50	0.08	*ASH*
1431	1431	Gurjunene <β->	0.73	0.89	1.47	0.78	*ASH*
1436	1440	Farnesene <(Z)-β->	0.54	0.13	-	-	*ASH*
1439	1449	Aromadendrene	0.37	0.19	0.56	0.10	*ASH*
1446	1448	Muurola-3,5-diene <*cis*->	-	-	0.45	0.36	*ASH*
1453	1452	Humulene <α->	0.48	0.12	1.20	0.47	*ASH*
1461	1452	*cis*-Cadina-1(6),4-diene	*-*	-	0.99	1.36	*ASH*
1471	1463	Dauca-5,8-diene	-	-	0.38	0.09	*ASH*
1475	1466	*trans*-Cadina-1(6),4-diene	*-*	-	0.99	0.12	*ASH*
1478	1479	Amorpha-4,7(11)-diene	-	-	0.15	0.07	*ASH*
1479	1479	Curcumene <ar->	5.06	2.01	-	-	*ARS*
1482	1478	Muurolene <γ->	0.41	0.01	0.92	0.23	*ASH*
1481	1481	Curcumene <γ->	52.02	8.62	-	-	*ARS*
1484	1484	Germacrene D	0.38	0.27	-	-	*ASH*
1486	1489	Selinene <β->	0.85	0.14	-	-	*ASH*
1492	1481	*cis*-β-Guaiene	*-*	-	0.71	0.11	*ASH*
1493	1486	Bicyclogermacrene	-	-	4.62	0.58	*ASH*
1493	1492	Selinene <δ->	-	-	0.81	0.08	*ASH*
1503	1505	Farnesene <(*E*,*E*)-α->	2.15	0.10	0.83	0.25	*ASH*
1510	1500	Muurolene <α->	-	-	0.91	0.17	*ASH*
1513	1505	Cadinene <γ->	0.35	0.09	2.86	0.37	*ASH*
1514	1508	Cubebol	-	-	0.36	0.21	*SOH*
1521	1512	*trans*-Calamenene	*-*	-	0.15	0.04	*ARS*
1522	1511	Cadinene <δ->	0.77	0.88	6.96	0.99	*ASH*
1521	1521	Sesquiphellandrene <β->	0.16	0.07	-	-	*ASH*
1533	1523	*trans*-Cadina-1,4-diene	*-*	-	0.37	0.10	*ASH*
1534	1537	Cadinene <α->	-	-	0.39	0.12	*ASH*
1538	1545	Selina-3,7(11)-diene	-	-	0.14	0.04	*ASH*
1567	1559	Germacrene B	3.10	2.29	0.18	0.06	*ASH*
1574	1567	Germacrene D-4-ol	-	-	1.46	0.40	*SOH*
1582	1569	Caryophyllene oxide	-	-	0.29	0.24	*OTH*
1577	1577	Spathulenol	0.62	0.23	-	-	*SOH*
1590	1584	Globulol	0.43	0.49	5.91	2.61	*SOH*
1592	1592	Viridiflorol	0.25	0.15	1.29	0.45	*SOH*
1594	1594	Carotol	0.89	0.49	-	-	*SOH*
1618	1617	1,10-di-*epi*-Cubenol	-	-	0.27	0.11	*SOH*
1618	1623	Junenol	-	-	1.39	0.42	*SOH*
1629	1622	Eudesmol <10-*epi*-γ->	-	-	0.54	0.15	*SOH*
1636	1639	Acorenol <β->	-	-	0.47	0.81	*SOH*
1639	1632	Acorenol <α->	-	-	Trace	-	*SOH*
1649	1644	Eudesmol <β->	0.24	0.05	4.47	1.93	*SOH*
1688	1681	Shyobunol	-	-	10.80	5.91	*SOH*
	*Aliphatic monoterpene hydrocarbons (AMH)*		10.83		24.50		
	*Aromatic monoterpene hydrocarbons(ARM)*		0.24		0.10		
	*Monoterpene alcohols (MOH)*		-		0.70		
	*Monoterpene ketones (MKE)*		5.57		-		
	*Aliphatic sesquiterpene hydrocarbons (ASH)*		14.03		35.80		
	*Aromatic sesquiterpene hydrocarbons(ARS)*		57.08		0.15		
	*Sesquiterpene alcohols (SOH)*		2.43		27.00		
	*Other compounds (OTH)*		1.90		3.86		
		Total identified	92.08		92.10		

^a^ Calculated retention indices according to van den Dool and Kratz [30]. ^b^ Reference retention indices according to Adams [31].
