# Supplementary material for: Acaricidal Efficacy of Plants from Ecuador, Ambrosia peruviana (Asteraceae) and Lepechinia mutica (Lamiaceae) against Larvae and Engorged Adult Females of the Common Cattle Tick, Rhipicephalus microplus
Source: Vet Sci. 2022 Jan 11;9(1):23. doi: 10.3390/vetsci9010023 (PMC8779275; doi:10.3390/vetsci9010023)
Supplement: Supplementary file 1 [file vetsci-09-00023-s001.zip › vetsci-1485393-supplementary/Figure S1. Ambrosia peruviana. Corrected..pdf]

Figure S1. Graph and data for calculation of LC_{50} and LC_{90} in larval package test with essential oil of *Ambrosia peruviana* (data obtained from Prism v9.3.0 for Windows, GraphPad Software, USA).

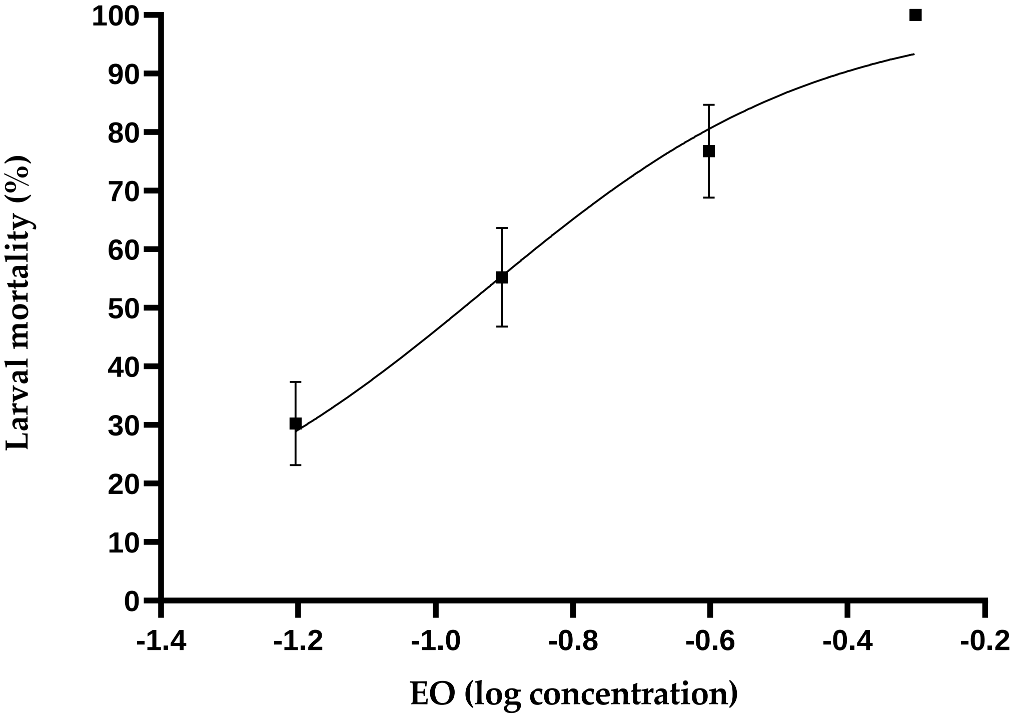

Basal value	6.00
Top value	100.00
logLC50	-0.9285
log LC90	-0.4113
Hill Slope	1.786
R^2	0.623

$$y = 6 + (100/(1 + 10\text{^}((\log CL - x) \times 1.786))$$
