# Supplementary material for: Acaricidal Efficacy of Plants from Ecuador, Ambrosia peruviana (Asteraceae) and Lepechinia mutica (Lamiaceae) against Larvae and Engorged Adult Females of the Common Cattle Tick, Rhipicephalus microplus
Source: Vet Sci. 2022 Jan 11;9(1):23. doi: 10.3390/vetsci9010023 (PMC8779275; doi:10.3390/vetsci9010023)
Supplement: Supplementary file 1 [file vetsci-09-00023-s001.zip › vetsci-1485393-supplementary/Figure S2. Lepechinia mutica. Corrected..pdf]

Figure S2. Graph and data for calculation of LC_{50} and LC_{90} in larval package test with essential oil of *Lepechinia mutica* (data obtained from Prism v9.3.0 for Windows, GraphPad Software, USA).

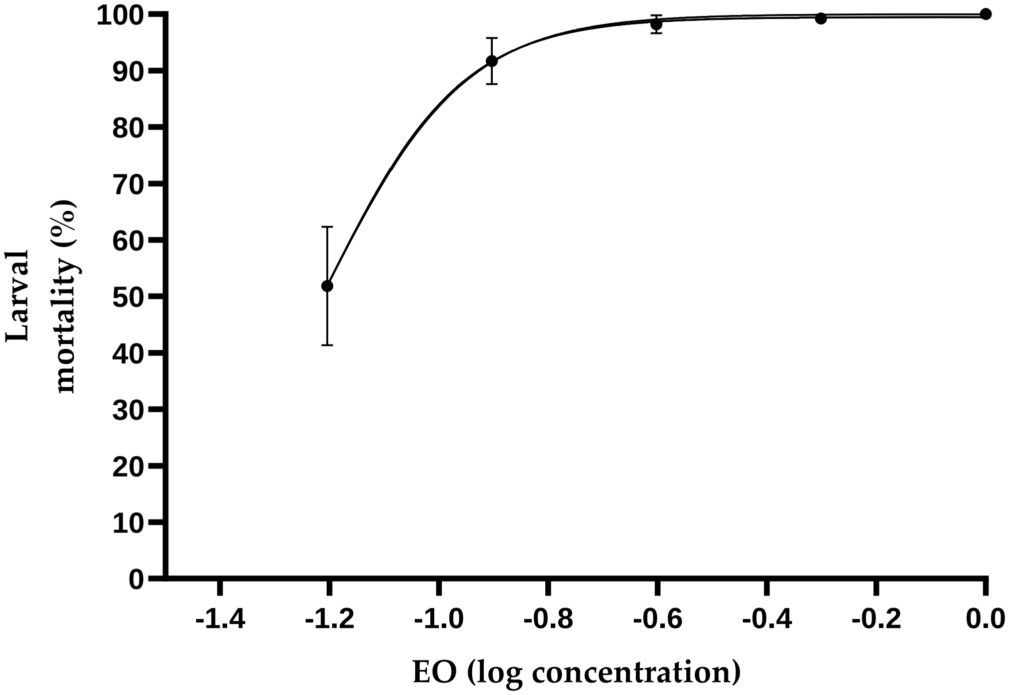

Basal value	5.128
Top value	100.00
logLC50	-1.200
logLC90	-0.925
Hill Slope	3.381
R^2	0.5916

$$y = 5.128 + (100/(1 + 10^{\wedge}((\log LC - x) \times 3.381))$$
