# Supplementary material for: Acaricidal Efficacy of Plants from Ecuador, Ambrosia peruviana (Asteraceae) and Lepechinia mutica (Lamiaceae) against Larvae and Engorged Adult Females of the Common Cattle Tick, Rhipicephalus microplus
Source: Vet Sci. 2022 Jan 11;9(1):23. doi: 10.3390/vetsci9010023 (PMC8779275; doi:10.3390/vetsci9010023)
Supplement: Supplementary file 1 [file vetsci-09-00023-s001.zip › vetsci-1485393-supplementary/Table S1.pdf]

Table S1. Mean and standard error (SE) of larval mortality values obtained by larval package test with essential oils of *Ambrosia peruviana* and *Lepechinia mutica*

EO (%)	Log	*Ambrosia peruviana*		*Lepechinia mutica*	
		**Mean**	**SE**	**Mean**	**SE**
0.625	-1.204	30.577	7.996	51.823	10.523
0.125	-0.903	54.227	9.901	91.687	4.071
0.250	-0.602	77.544	9.527	98.2131	1.570
0.5	-0.301	100	0	99.2536	0.336
1	0	-	-	100	0
